# Benefits of Vitamin D in Health and Diseases

**DOI:** 10.3390/nu15112419

**Published:** 2023-05-23

**Authors:** Giovanni Passeri, Sandro Giannini

**Affiliations:** 1 Unit of Clinica e Terapia Medica, Department of Medicine and Surgery, University of Parma, 43126 Parma, Italy; 2 Clinica Medica 1, Department of Medicine, University of Padova, 35128 Padova, Italy; sandro.giannini@unipd.it

This Special Issue of *Nutrients*, titled “Benefits of Vitamin D in health and diseases”, includes a total of twenty-five publications that consider different aspects of vitamin D, both at the cellular/preclinical and clinical levels, in neonates or children, in pregnant women, in adults and in elderly subjects. This Special Issue consists of nineteen original articles, five reviews and one systematic review.

Vitamin D is a micronutrient and an endogenous metabolite acting as a hormone, binding to the vitamin D receptor (VDR) present in many organs and tissues. It has a pivotal role in the maintenance of bone and muscular health and immune function and is able to directly or indirectly regulate several metabolic pathways.

Vitamin D status is best estimated through serum 25-hydroxy vitamin D (25(OH)D) concentration, but this measurement remains complex, and different methods are still in use for its evaluation in either serum or in plasma [1]. The standardization of 25(OH)D measurements is an ongoing process led by the International Federation of Clinical Chemistry and Laboratory Medicine (IFCC), the Vitamin D Standardization Program (VDSP) and the Centers for Disease Control (CDC) Vitamin D Standardization Certification Program (VDSCP) [2]. The biological variation (BV) of 25(OH)D was evaluated in six European laboratories for up to 10 weeks using a non-competitive immunoassay (sandwich) method in order to set analytical performance specifications (APS) for measurement uncertainty (MU). It was demonstrated that the APS recommended by the clinical laboratory communities and developed by Stöckl et al. more than a decade ago [3] can evolve to include MU [4].

Vitamin D deficiency (VDD) affects a high percentage of individuals worldwide. In a rural setting in Nepal, where no food is fortified and diets contain only scarce amounts of vitamin D, but sun exposure can be considered overall sufficient, a high prevalence of hypovitaminosis D among women was observed. Age was an important predictor of vitamin D status, and VDD was associated with high glycated hemoglobin (HbA1c) and dyslipidemia, implying a possible role of VDD in the pathogenesis of metabolic syndrome (MS) [5]. In Italy, some restrictions on the reimbursement criteria for vitamin D prescriptions have been applied in order to limit excessive and, in some cases, not necessary supplementation, especially in young healthy adults. In this context, Degli Esposti et al. highlighted that a possible misinterpretation of Italian criteria for reimbursement restrictions in vitamin D outside of osteoporosis have resulted in an inadequate level of vitamin D supplementation in patients with osteoporosis, and undertreatment can reduce the effect of osteoporosis therapies, leading to increased risk of negative outcome [6]. Interestingly, with the aim of identifying adults at risk of vitamin D insufficiency and avoiding unwarranted supplementation or blood testing to target and treat at-risk individuals, the EVIDENCe-Q project (Evaluation VItamin D dEficieNCy Questionnaire) was proposed. This simple method is an easy-to-use questionnaire that demonstrates a good relationship with the gold standard vitamin D level, showing potential for good decision-making support in clinical practice for screening for vitamin D deficiency in asymptomatic adults [7].

There is still much to be debated about the relationship between maternal 25(OH)D status and bone outcomes in infancy [8] or regarding gestational weight gain [9]. It was demonstrated in one of the articles published in this Special Issue [10] that maternal serum 25(OH)D predicts neonatal 25(OH)D but was not related to neonatal bone mass of healthy-term, appropriate for gestational age infants born to predominantly vitamin D sufficient mothers. It was also acknowledged that more detailed evidence is necessary to elucidate the impact of insufficient maternal vitamin D status on offspring’s bone outcomes.

The association between vitamin D status or intake and mental health in children was considered in a systematic review [11]. The importance of meeting the required 25(OH) cholecalciferol blood level to prevent or alleviate mental health problems in children was demonstrated. Therefore, vitamin D obtained from a balanced diet or via supplementation was indicated as an element supporting mental health in childhood [11]. In children with autism spectrum disorder (ASD), significantly lower vitamin D levels than normally developing children have been consistently reported, and interestingly, vitamin D deficiency was found to be strongly correlated with ASD severity [12]. In a review focused on the role of vitamin D supplementation in children with ASD, it was observed that improved vitamin D status significantly reduced the severity of ASD. However, due to common limitations, such as a small number of participants and short duration of follow-ups in the selected studies, this effect was not consistently different between the treatment and control groups. The variations in vitamin D dose protocols, the presence of concurrent interventions and the age of the child when the vitamin D intervention was introduced were identified as possible factors determining the effectiveness of the treatment [13].

The association between VDD and cardiometabolic diseases has been intensively studied, but definitive causal effects have not been established yet [14]. A genetic approach able to provide a better understanding of this potential association was considered in Turkish adults. A novel interaction between metabolic genetic risk score (GRS) and dietary fat intake on serum vitamin D concentrations was demonstrated, suggesting that following current dietary fat intake recommendations (<35%) might be effective in preventing any consequences of the genetic risk of VDD [15]. Considering the role of vitamin D during diabetes development, epidemiological studies have suggested that patients with impaired fasting glucose (IFG) and/or impaired glucose tolerance (IGT) have lower serum 25(OH)D levels [16]. In adult subjects with prediabetes, oral supplementation of vitamin D was shown to exert better effects in improving fasting blood glucose, HbA1c and fasting insulin compared with controls, while no difference was found in BMI. Moreover, long-term vitamin D supplementation in patients with vitamin D deficiency showed an additional beneficial effect within 2 h via oral glucose tolerance test plasma glucose, in insulin resistance via homeostasis model assessment and in homeostasis model assessments of b-cell function [17]. A possible correlation between obesity and vitamin D deficiency has already been suggested, and low vitamin D is consistently found in obese subjects across age groups, ethnicities and geography. However, data connecting obesity, low vitamin D levels and the development of MS are very limited and present conflicting results. In individuals with severe obesity (class II and III obesity with BMI ≥ 35 kg/m^2^ and ≥40 kg/m^2^, respectively) the association of serum and dietary vitamin D with MS and its parameters was tested. Surprisingly, severely obese subjects showed low prevalence of vitamin D deficiency, which was not associated with MS. Serum and dietary vitamin D were also not associated with MS, except for low HDL cholesterol levels, which were a protective factor for serum vitamin D deficiency [18]. Myostatin is a myokine produced by muscle that prevents muscle anabolism. In a group of severely obese child and adolescent patients, an independent negative correlation was observed between low vitamin D levels and elevated myostatin [19].

Three different articles published in this Special Issue considered specific aspects of the association between vitamin D deficiency (VDD), cardio-vascular risk and metabolic diseases. In an elegant study by Sipos M et al., vitamin D supplementation in a vitamin D-deficient murine model was studied by comparing male and female rats on an important hypertension target organ, the renal artery. It was confirmed that vitamin D deficiency in both sexes increases cardiovascular risk, and in particular, a vascular relaxing dysfunction with decreased renal perfusion was present in the long term, affecting the function of the renin–angiotensin–aldosterone system that can contribute to further vascular dysfunction and hypertension. This effect was more evident in female rats, which showed more advanced changes [20]. The importance of normal 25(OH) D serum levels has been shown during different physically stressful conditions. In patients after laparoscopic one-anastomosis gastric bypass (OAGB), preoperative level of 25(OH)D above 30 ng/mL were related to beneficial post-effects of bariatric surgery, especially regarding the lipid profile. A decrease in Triglycerides and CRP with HDL-cholesterol was noted, irrespective of vitamin D status, while in patients with 25(OH)D below 30 ng/mL, total cholesterol was significantly increased [21]. The different behaviors of vitamin D supplementation was studied comparing vitamin D-deficient black versus white individuals living in the Southern Sahara (SS) affected by chronic kidney disease (CKD). It was highlighted that the severity of vitamin D deficiency depends on racial–ethnic factors, and that is more acute in black SS CKD patients than in white patients. Overall, despite different doses and times needed in the two populations to reach normal 25 (OH)D levels, vitamin D supplementation achieved beneficial effects such as reduced pro-inflammatory cytokine storm and oxidative stress damage and prevention of its progression to end-stage renal failure. On the other hand, a reduction in athero-thromboembolic risk related to vitamin D supplementation was not evident in this clinical setting, particularly in black SS residents [22].

Vitamin D status is associated with muscle strength, and low 25(OH)D levels have been related with poor physical performance in older adults. The debate on the potential effect of vitamin D supplementation on physical activity is still ongoing. Aschauer R. et al. [23] evaluated whether vitamin D supplementation can provide an additive effect to muscular training to ameliorate physical performance. In a group of community-dwelling healthy elderly subjects with basal 25 (OH)D levels above 20 ng/mL, a supplementation of either 800 UI daily (VDd) or 50.000 UI monthly (VDm) was able to increase 25 (OH) vitamin D by less than 10 ng/mL as compared to control subjects after 17 weeks of treatment. In this specific setting, using a moderate supplementation of cholecalciferol for less than 6 months, no additive effects on resistance training from either VDd or VDm were observed. To testify to the complexity of the interaction between vitamin D levels and physical activity, in a different clinical setting (adults with cerebral palsy) with levels of 25(OH)D in the range of insufficiency (on average 17 ng/mL), a positive association between 25(OH)D level and specific muscle function was found in these patients but not in the physical-activity-matched controls without neurological impairment. The authors therefore suggest vitamin D supplementation at least during the winter months to ensure that decreased muscular performance and potential risk of falls from exacerbated knee extensor weakness is reduced [24].

Vitamin D, a hormone acting as a nutrient, exerts its activity by binding to a specific nuclear receptor called the vitamin D receptor (VDR). Three articles published in this Special Issue underline the importance of the VDR expression and gene polymorphism in different physiological or pathological conditions. Vitamin D, mediated through the vitamin D receptor (VDR), has been identified as a protective nutrient against ionizing radiation (IR)-induced damage. In a murine model, it was investigated whether VDR could inhibit IR-induced intestinal injury, and the underlying mechanism was explored. Indeed, vitamin D induced VDR expression and inhibited IR-induced DNA damage and apoptosis in vitro. VDR was shown to be highly expressed in intestinal crypts and was critical for crypt stem/progenitor cell proliferation under physiological conditions. In VDR-deficient mice exposed to IR, DNA damage and crypt stem/progenitor cell apoptosis significantly increased, leading to impaired intestinal regeneration, as well as shorter survival time. VDR regulated the Pmaip1-mediated pathway, diminished stem/progenitor cell apoptosis and further protected against intestinal injury induced by IR, suggesting that the VDR might be a potential target for prevention and management of IR-induced intestinal injury [25]. Childhood stunting remains a major public health problem in developing countries. Micronutrient intake, such as calcium and vitamin D, play an important role in growth of children, and the VDR gene is involved in calcium absorption. In a group of elementary school children aged 8–10 years old in East Java, the association between VDR gene polymorphism and dietary intake towards height-for-age z-score (HAZ) was studied. Two single-nucleotide polymorphisms in the promoter region of VDR gene were considered, and a significant correlation was shown between energy and protein intake with the HAZ of the children; however, no association between the VDR gene and the HAZ was found. Genotype analysis also showed that Indonesian children had a favorable VDR gene genotype; however, the effect of VDR gene promoter activity on HAZ might not be revealed due to very low vitamin D intake to stimulate intestinal calcium absorption, which in turn affects the HAZ of the children [26]. An association between low systemic levels of vitamin D, poor breast cancer prognosis and expression of the vitamin D receptor (VDR) in breast cancers and survival has been suggested. Huss L. et al. studied the associations between pre-diagnostic systemic levels of vitamin D and the expression of the VDR in subsequent breast tumor patients, as well as the interactions between vitamin D and the VDR on breast cancer mortality [27]. In women with high levels of vitamin D, a smaller proportion of VDR-negative breast tumors was found as compared to women with low levels of vitamin D. Moreover, vitamin D levels were not found to modify the association between low VDR expression and high breast cancer mortality. No statistical evidence for an association between pre-diagnostic levels of vitamin D and the expression of VDRs in breast cancer was found, nor did vitamin D levels influence the association between VDR expression and breast cancer mortality.

As the main and well-recognized functions of vitamin D are the regulation of calcium homeostasis and the maintenance of a healthy mineralized skeleton, there is growing evidence that this hormone has an important role in different functions such as tumor suppression, anti-inflammation and immune regulation. Regarding malignant tumors, vitamin D insufficiency (VDI) and vitamin D deficiency (VDD), together with urban dwelling and overweight and obesity, have been suggested as possible factors in developing sporadic retinoblastoma (SRb) in children, as evidenced in the analysis of data from the Epidemiology of SRb in Mexico (EpiRbMx) study and the National Health and Nutrition Survey 2018–2019 (ENSANUT 2018–2019) [28]. Uterine myomas or leiomyomas, also called uterine fibroids (UFs), are monoclonal tumors derived from the myometrium and are the most frequent benign tumors in women of reproductive age [29]. The correlation between vitamin D levels and UFs, the mechanisms underlying the action of vitamin D in preclinical or clinical settings and the possible role of vitamin D in UFs were analyzed in a review published in this Special Issue. Interesting positive results in vitro or in preclinical models of UFs demonstrated that vitamin directly or indirectly regulated gene expression and modulation of intracellular signaling pathways. However, these promising suggestions failed to translate into convincing and definitive clinical evidence since the complex genomic profile of UFs or the presence of multiple extracellular stimuli may influence vitamin D signaling [30]. It has been shown that vitamin D plays a critical role in the regulation of inflammation in pancreatitis, and its anti-inflammatory and anti-fibrotic effects are exerted by binding with the vitamin D receptor (VDR). Vitamin D deficiency has been linked with increment in inflammatory cytokines, bone damage and glucose metabolism during both acute (AP) and chronic pancreatitis (CP). Interventional studies did not demonstrate an unequivocally beneficial effect of vitamin D supplementation in pancreatitis, but some encouraging results have emerged from trials on patients at risk of developing complications related to pancreatic inflammation, suggesting a promising potential of vitamin D supplementation to address inflammatory and fibrotic diseases [31]. In addition to its well-known anti-inflammatory properties, vitamin D is involved in immune-mediated disorders, such as inflammatory bowel disease (IBD). In children with IBD, a complex analysis of factors affecting vitamin D status was performed, suggesting that in pediatric patients, regular monitoring of serum vitamin D level and its adequate supplementation is highly advisable for both IBD and healthy individuals [32].

Due to its immunomodulating properties, vitamin D status and supplementation has been widely considered in COVID-19 patients in the past three years. In particular, an interesting study considered the protective effect of vitamin D supplementation on the development of severe forms of COVID-19 in older adults. Vitamin D supplementation taken before the onset of the disease seems to have a protective effect on the development of severe forms of COVID-19 in patients aged over 70 years, although no relationship between serum 25(OH)D levels and the severity of SARS-CoV-2 infection was demonstrated [33].

Finally, a narrative review considered the available evidence from both RCTs and real-life observational studies on the potential role of vitamin D in selected immune-mediated diseases, such as rheumatic diseases and diabetes, and infectious diseases, including COVID-19. A generalized reduction in circulating 25(OH)D serum levels in these patients compared to healthy individuals was observed. Moreover, outcome measures were improved in these clinical settings following vitamin D supplementation, particularly at high and daily doses in patients already deficient in vitamin D. There was also some evidence of modest or absent efficacy of vitamin D therapeutic supplementation, probably due to the presence of several confounding variables such as the recruitment of not only vitamin D-deficient patients, the different metabolite supplemented (i.e., either “native” vitamin D or 25(OH)D or calcitriol) and the dose, age of the patients, study duration, high BMI and baseline comorbidities [34].

Overall, considering the involvement of vitamin D in the direct or indirect regulation of over one thousand genes in humans, there is still a need for large RCTs in order to confirm whether sufficient vitamin D intake can maintain a physiological healthy status and can reduce the incidence and severity of clinical conditions such as infections, inflammatory and autoimmune diseases, neuromuscular complications and cancer.
